# Antioxidant and DNA-Protective Activity of an Extract Originated from Kalamon Olives Debittering

**DOI:** 10.3390/antiox12020333

**Published:** 2023-01-31

**Authors:** Maria Kourti, Maria V. Alvanou, Zoi Skaperda, Fotis Tekos, Georgios Papaefstathiou, Panagiotis Stathopoulos, Demetrios Kouretas

**Affiliations:** 1 Laboratory of Animal Physiology, Department of Biochemistry-Biotechnology, School of Health Sciences, University of Thessaly, 41500 Larissa, Greece; 2 Laboratory of Animal Molecular Genetics, Department of Agriculture, University of Western Macedonia, 50100 Kozani, Greece; 3 PharmaGnose S.A., Papathanasiou 24, 34100 Chalkida, Greece; 4 Department of Pharmacognosy and Natural Products Chemistry, Faculty of Pharmacy, University of Athens, 15771 Athens, Greece

**Keywords:** brine extract, Kalamon olives, biophenols, antioxidant activity, DNA-protection

## Abstract

Table olives are a major component of the Mediterranean diet and are associated with many beneficial biological activities, which are mainly related to their phenolic compounds. Olive fruit debittering process defines the quantitative and qualitative composition of table olives in biophenols. The aim of the present study was to evaluate the in vitro antioxidant capacity and DNA-protective activity of an extract originated from brine samples, according to the Greek style debbitering process of Kalamon olive fruits. The main phenolic components determined in the brine extract were hydroxytyrosol (HT), verbascoside (VERB) and tyrosol (T). The in vitro cell-free assays showed strong radical scavenging capacity from the extract, therefore antioxidant potential. At cellular level, human endothelial cells (EA.hy296) and murine myoblasts (C2C12) were treated with non-cytotoxic concentrations of the brine extract and the redox status was assessed by measuring glutathione (GSH), reactive oxygen species (ROS) and lipid peroxidation levels (TBARS). Our results show cell type specific response, exerting a hormetic reflection at endothelial cells. Finally, in both cell lines, pre-treatment with brine extract protected from H_2_O_2_-induced DNA damage. In conclusion, this is the first holistic approach highlighted table olive wastewaters from Kalamon- Greek style debittering process, as valuable source of bioactive compounds, which could have interesting implications for the development of new products in food or other industries.

## 1. Introduction

*Olea europaea* L. cultivation characterizes the agricultural production of all the Mediterranean countries. Olive fruits are mainly used to produce oil and table olives, products that are an integral part of the Mediterranean diet, which have been associated with many health benefits in humans [1,2,3,4,5]. In Greece, Kalamon olive variety is among the most famous olive cultivars, which is better known as Kalamata, from the name of a town in Greece, where many of these olives are grown. These olive fruits most often are eaten as black table olives, but they can also be used to make olive oil [1,3,4,5,6].

Olive fruits contain a variety of biophenols. The main phenolic component is oleuropein, responsible for the bitter taste of olive fruits, which must be removed during table olive processing, in order to make the olive fruit more palatable [7]. There are three main types of olive fruit debittering: (a) treated green olives (or Spanish style); (b) olives darkened by oxidation (Californian style); (c) natural (mainly black) olives (Greek style) [8]. The debittering process is very important, as it defines the quantitative and qualitative composition in phenolic compounds of table olives, thereby changing their nutritional and healthy properties [8,9].

For the Greek style debittering process, olives close to full ripeness (purple-black color) are used. The olives are washed and placed in a brine solution (8–10% *w/v* NaCl), where the bitter taste of the phenolic compounds is removed by diffusion from the fruit [8]. Oleuropein, the main phenolic compound in olives, is hydrolyzed through β-glucosidase and esterase enzymes during debittering, from the microbiota present in the brine or on the surface of the olive fruit. The major products of oleuropein hydrolysis, into the brines, include HT and T [10]. Depending on the type of debittering procedure, a different style of table olives is produced, with a unique texture and chemical, microbial, and sensorial profiles [8]. Comparing fermented table olives resulting from the three debittering types, studies have shown that table olives according to Greek style have the highest content in polyphenols, a fact that shows a high nutritional value [8,11].

The wastewaters of the debittering process are disposed without prior treatment, a fact that has led to severe ecological problems. More specifically, table olive wastewaters (TOWW), with polyphenolic content up to 10 g/L, exerted toxicity to some plants and microorganisms [12]. However, only a few studies have investigated the composition and the bioactivity of the brine solution from the olive table processing [9,10,13,14,15,16,17]. The recovery of bioactive metabolites, especially HT, aromatic acids, and conjugated aromatic acids from brines, is of particular interest due to very promising bioactivities and health promoting properties [17,18].

In the present study, we applied a holistic in vitro approach to explore the antioxidant and geno-protective activity of an extract originated from brine samples, according to the Greek style debbitering process of Kalamon olive fruits. For this purpose, we applied five in vitro antioxidant cell-free assays, DPPH^•^, ABTS^•+^, O_2_^•−^, RP and ROO• and we estimated ROS, GSH and TBARS levels in two cell lines. Also, we investigated the ability of the brine extract to protect from DNA damage under oxidative conditions, using Comet assay.

## 2. Materials and Methods

### 2.1. Raw Materials

The raw materials of edible olive debittering water were obtained from a collaborating edible olive processing factory located in Evia region of Greece. Wastewater was produced during the Greek-style debittering process of Kalamon olives and was collected during the period 2019–2020. When the Greek style is used, the black olives are placed directly in brine without any NaOH treatment and left there for months in order to remove the bitterness completely. These side-products resulting from the debittering process of Kalamon black olives were used as raw material for the production of the enriched extracts, tested in this study.

### 2.2. Production of Enriched Extracts from Table Olive Wastewater (TOWW)

1000 kg of Table Olive Wastewater (TOWW) was filtered to remove all the suspended particles using a sequential filtering process comprised of different size filters: 250, 100, 50, 25 and 10 micros. Then, 900 kg of the clarified material was obtained with a total solid content of 14.5% (Kern DBS Moisture analyzer). The increased TS value was due to the high concentration of NaCl present in table olive processing water, since it was used as debittering agent. Excess salt and other non-active ingredients were removed using adsorption resin technology. Specifically, a column of 70 cm in diameter and 2 m height, filled with 450 lt of XAD 4 resin, was used and then, the filtered table olive processing water passed through the column using a diaphragmatic pump with a flow of 2.5 kg/min. The procedure was monitored with TLC using dichloromethane:methanol 90:10 (*v/v*) as mobile phase. After completion of the absorption process, the resin was washed with 100 kg of water. The desorption and the complete recovery of the phenolic components from the resin was carried out by passing a solvent mixture of 800 kg Water/Isopropanol: 1/1 (*v/v*), through the column, with a flow rate of 2.5 kg/h.

Afterwards, the obtained extract was evaporated under reduced vacuum initially in a QVF 200 lt glass evaporator and subsequently in a 20 lt Buchi rotary evaporator. The evaporation process was completed when the weight of the concentrated extract was reduced to 110 kg and specifically when the T.S. reached 5.67%, (ideal T.S. range between 5 and 15%). The formulation of the extract in powder form was performed after the addition of maltodextrin D.E. 12 in the concentrated extract, at a rate of 75% of the total solids (weight 4677 g), applying the Spray dryer technique. Specifically, 4.6 kg of maltodextrin 12 D.E. were dissolved in 9 kg of distilled water and added in the concentrated extract, prior to drying. The applied spray drying parameters that tested were: inlet temperature 170 °C, outlet temperature 80 °C, spraying pressure 1.5 bar, nozzle diameter 0.5 mm and pump flow 4 lt/h. At the end of the procedure 9.9 kg of dry powder was collected with a spray dryer efficiency of 91% and an obtained powder with a good particle size, flowing properties, color, and texture.

### 2.3. HPLC-DAD Analysis and Quantification

The determination of olive biophenols in extract of TOWW was performed by the IOC proposed analytical method. The IOC proposed method was performed according to analytical conditions referred to IOC/T.20/Doc No 29 method [19]. Specifically, the separation was achieved on a reversed-phase Spherisorb Discovery HS C18 column (250 × 4.6 mm, 5 μm; Supelco) using a mobile phase consisting of 0.2% aqueous orthophosphoric acid (A) and MeOH/ACN (50:50 *v/v*) (B), at a flow rate of 1.0 mL/min and ambient temperature. The injection volume was held constant at 20 μL. The applied gradient elution was as follows: 0 min, 96% A and 4% B; 40 min, 50% A and 50% B; 45 min, 40% A and 60% B; 60 min, 0% A and 100% B; 70 min, 0% A and 100% B; 72 min, 96% A and 4% B; 82 min, 96% A and 4% B. Chromatograms were monitored at 280 nm. All analyses were made in triplicate.

Concentration levels of major biophenols have been also determined using regression analysis method. Specifically, standard calibration curves of Hydroxytyrosol (HT), Tyrosol (T), and Verbascoside (VERB) were prepared. For the HT and T quantification, 9-points calibration curves were constructed (HT: y = 84,028x + 39,609, r^2^ = 0.9997; T: y = 53,933x − 11,712, r^2^ = 0.9987), while VERB quantified according to 5-points calibration curve, respectively (VERB: y = 71488x − 50153, r^2^ = 0.9997). HT, T and VERB reference standards were purchased from ExtraSynthase (Lyon Nord, France).

### 2.4. In Vitro Cell-Free Methods

All chemicals for the following assays were purchased from Sigma-Aldrich, Munich, Germany.

#### 2.4.1. 2,2-Diphenyl-1-Picrylhydrazyl (DPPH) Radical Scavenging Assay

The DPPH^•^ assay was evaluated as described previously [20,21]. Briefly, 100 μM DPPH^•^ radical (1.0 mL) diluted in methanol was mixed with increasing concentrations of the brine extract, in triplicates. After a 20 min incubation in the dark at room temperature (RT), the absorbance was read at 517 nm on a Hitachi U-1900 radio beam spectrophotometer (serial no. 2023-029; Hitachi). Methanol alone was used as blank, while DPPH^•^ radical alone in methanol was used as control, in each experiment. Then, according to the following equation:

RSC (%) = (A_control_ − A_sample_)/A_control_× 100

the % of radical scavenging capacity (RSC) of the samples was calculated, where A_control_ and A_sample_ are the absorbance values of the control and the test sample, respectively. The results were expressed as IC_50_ value, indicating the sample concentration that causes 50% scavenging of DPPH^•^ radical, which was estimated from the graph-plotted %RSC against the sample concentration. Ascorbic acid was used as a positive control.

#### 2.4.2. ABTS^•+^Radical Scavenging Assay

The 2,2′ -Azino-bis-(3-ethyl-benzthiazoline-sulphonic acid) (ABTS) radical cation (ABTS^•+^) decolorization assay was determined according to the method described by Cano et al. (1998) with some modifications [20,21]. In brief, triplicates were prepared for each concentration, containing the ABTS^•+^ radical, which produced by mixing 500 μL of ABTS (1 mM), 50 μL H_2_O_2_ (30 μM), 50 μL horseradish peroxidase (6 μM) and 400 μL dionized water (dH_2_O). Next, the reaction tubes incubated in the dark at RT, for 45 min. Then, 50 μL of increasing extract concentrations were added in the reaction mixture and the absorbance was monitored at 730 nm (serial no. 2023-029; Hitachi). As blank, a solution without the peroxidase was used, while as control the ABTS^•+^ radical solution without the sample was used. The RSC (%) and the IC_50_ values were determined as described for the DPPH^•^ assay. Ascorbic acid was used as a positive control.

#### 2.4.3. Superoxide (O_2_^•−^) Radical Scavenging Assay

Superoxide (O_2_^•−^) radical scavenging activity was determined according to the method of Gülçin et al. (2004) [22]. Briefly, 625 μL Tris–HCl (16 mM, pH 8.0), 125 μL of NBT (100 μM), 125 μL of NADH (468 μM) and 50 μL of extract at increasing concentrations were mixed, in triplicates. 125 μL of PMS (60 μM) was added and the tested tubes were incubated for 5 min and centrifuged at 3000 rpm for 10 min at 25 °C. Next, the absorbance was measured at 560 nm (serial no. 2023-029; Hitachi). In each experiment, the samples without PMS were used as blanks and the samples without extract were used as controls. The RSC (%) and the IC_50_ values were determined as described above for the DPPH^•^ assay. Instead of ascorbic acid, ellagic acid was used as positive control.

#### 2.4.4. Reducing Power Capacity

The assay was performed according to protocol of Yen and Duh, (1993) [23], with some modifications [21]. In brief, 200 μL phosphate buffer (0.2 M, pH 6.6) and 250 μL of potassium ferricyanide (1% *w/v*) were mixed with 50 μL of increasing concentrations of brine extract, in triplicates. Then, the reaction tubes were incubated at 50 °C for 20 min and were cooled on ice for 5 min. Next, 250 μL of TCA (10% *w/v*) was added and the samples were centrifuged at 3000 rpm for 10 min at 25 °C. Following, reaction tubes containing 700 μL of the supernatant, 250 μL of deionized water and 50 μL ferric chloride (0.1% *w/v*) were incubated for 10 min at RT. The results expressed as AU_0.5_ value, which was calculated from the graph-plotted absorbance against the extract concentration, indicating the extract concentration that causes an absorbance of 0.5 at 700 nm. Ascorbic acid was used as positive control.

#### 2.4.5. Peroxyl Radical-Induced DNA Strand Cleavage Assay

The assay was performed according to a previously described protocol [24] with some modifications as reported by Priftis et al. (2017) [25]. Briefly, 3.2 μg pBluescript-SK+ plasmid DNA was treated or not (negative controls) with 95 mM 2,2′-azobis (2-amidinopropane hydrochloride) (AAPH) in PBS, in the presence or not of increasing concentrations of the brine extract (6.25–200 μg/mL), in the dark for 45 min at 37 °C. In short, the samples were: sample 1: plasmid alone, sample 2: 95 mM AAPH alone, samples 3–8: 95 mM AAPH and 6.25–200 μg/mL of the brine extract and sample 9: 200 μg/mL of brine extract alone. At the end of incubation period, a 3 μL loading buffer was added and the samples analyzed by electrophoresis on a 0.8% *w/v* agarose gel at 80 V for 55 min. After staining with ethidium bromide, gels were exposed to UV and images were taken using MultiImage Light Cabinet and analyzed by an Image analysis quantification software. Each experiment was carried out at least three times.

The protective effect of the brine extract from ROO^•^-induced plasmid strand breakage was estimated, by measuring the inhibition of conversion of supercoiled conformation to open circular and linear forms, according to the following equation:

% inhibition = (S − S_o_)/(S_control_ − S_o_) × 100

where S_control_: % supercoiled DNA in plasmid DNA alone (negative control), S_o_: % supercoiled DNA in the plasmid DNA with AAPH alone (positive control), and S: the % supercoiled DNA in the presence of the tested extract and AAPH. IC_50_ values express the concentration needed to inhibit relaxation of supercoiled conformation induced by peroxyl radicals by 50%. Ascorbic acid was used as a positive control.

### 2.5. Cell-Based Assays

Cell culture

EA.hy926 endothelial cells and C2C12 murine myoblasts were cultured in 1 g/L glucose and 4.5 g/L glucose Dulbecco’s modified Eagle’s medium (DMEM), respectively, containing 10% (*v/v*) fetal bovine serum (FBS) and 100 U/mL of penicillin/streptomycin, at 37 °C, in 5% CO_2_ [26]. Cell culture materials were purchased from Gibco, Thermo Fisher Scientific, Waltham, MA, USA.

#### 2.5.1. XTT Assay

Cell viability was assessed using the XTT assay kit (Roche, Mannheim, Germany), according to manufacturer’s instructions. Briefly, EA.hy926 cells and the C2C12 cells were seeded into 96-well plate in a density of 10 × 10^3^ and 5 × 10^3^ cells per well, respectively, in complete DMEM. Next day, cells were treated with increasing concentrations of the brine extract in serum-free medium, for 24 h. Then, 50 μL of XTT test solution were added to each well and incubated for 4 h. Following, the absorbance was measured at 450 nm and at 630 nm as a reference wavelength in a microplate reader (BioTek Instruments, Inc., Winooski, VT, USA). Serum-free DMEM was used as a negative control, as well as the absorbance of the extracts alone in each concentration was read in serum-free medium, which were subtracted from the corresponding incubations in the cells.

Cell proliferation (%) = [(OD_control_−OD_sample_)/OD_control_] × 100,

where OD_control_ and OD_sample_ indicate the OD of untreated and treated cells, respectively. All experiments were carried out in triplicate and on three separate occasions.

#### 2.5.2. Flow Cytometry for ROS and GSH Detection

The intracellular ROS and GSH levels were assessed by flow cytometry, using DCF-DA and Intracellular glutathione (GSH) Detection Assay Kit (ab112132; Abcam, Cambridge, UK), respectively. 200,000 cells/well were seeded in two separate 6-well plates, one for ROS and one for GSH detection. After a 24 h treatment with increasing concentrations of the brine extract, the medium was removed and 1 mL of 10 μΜ DCF-DA (20 mM stock in DMSO) in PBS was added in each well of the ROS plate, and incubated in the dark at 37 °C for 45 min. After this incubation period, cells were trypsinized, centrifuged (1200 rpm, 5 min, 4 °C) and resuspended in 250 μLPBS. At the same time, when the 24 h treatment period was ended, cells of the GSH plate were trypsinized immediately, centrifuged (1200 rpm, 5 min, 4 °C) and GSH detection was performed according to the manufacturer’s instructions. Briefly, cells were resuspended in 1 mL PBS with 5 μL of Green dye and incubated in the dark at 37 °C for 30 min. After this, the cells were washed with 1 mL PBS to remove the excess dye, centrifuged (1200 rpm, 5 min, 4 °C) and resuspended in PBS. The cells were then submitted to flow cytometric analysis using a FACScan flow cytometer (Becton Dickinson, NJ, USA) with excitation and emission length at 490/520 nm for ROS and for GSH detection.

#### 2.5.3. TBARS Determination

After 24 h treatment with brine extract in medium without FBS, cells were washed with PBS and were detached from 25-cm^3^ flasks using a cell scraper in 250 μL PBS containing a Roche cOmplete™ protease inhibitor cocktail tablet (Roche Diagnostics, Mannheim, Germany). Then, the cells were lysed by periodical ultrasonication (70% amplitude, 0.5 s pulse cycle) on ice, for 10 s with a 10 s pause using an ultrasonic processor (UP400S, Hielscher, Teltow, Germany). This step was repeated 5 times in total. A centrifugation (15,000× *g*, 20 min, 4 °C) was followed and the supernatant was collected. Here in, protein concentration was quantified by Bradford assay [27], using a standard curve of bovine serum albumin, and 70 μg of total protein was used for each sample, in duplicate. In total 400 μL of PBS with or without cell lysate (blank), 500 μL of Tris-HCl (200 mM, pH = 7.4) and 500 μL of 35% TCA were added and the samples were incubated for 10 min at RT. Next, 1 mL of 2 M Na_2_SO_4_ and 55 mM TBA solution was added, and the samples were placed in water bath for 45 min at 95 °C. After incubation, the samples were cooled on ice for 5 min, 1 mL of 70% TCA was added, and the samples were centrifuged (11,200× *g*, 3 min, 25 °C). Then, the absorbance was measured at 530 nm. TBARS levels were calculated as the molar extinction coefficient of malondialdehyde (ΜDA) (156,000 L/mol/cm) and the results were presented as % fold change of control.

#### 2.5.4. Alkaline Comet Assay

Comet assay is a rapid, sensitive and relatively simple electrophoresis technique that measures DNA damage at single eukaryotic cells. The alkaline Comet, which we applied, is more sensitive in detecting DNA double and single stranded fragments and alkali labile sites [28]. The assay was performed as previously described [29], with some modifications. Briefly, the cells were seeded into 6-well plates (2 × 10^5^ cells/well) and were incubated overnight at 37 °C, in 5% CO_2_. Next, the cells were pre-treated at 40 μg/mL of brine extract for 24 h. 1 h before the end of 24 h incubation, 250 µM H_2_O_2_ was added, with and without the extract. Then, the cells were detached with trypsin-EDTA and washed with ice-cold PBS once. Cells were measured using Neubauer, and a suspension of 1 × 10^6^ cells/mL in PBS was prepared. Then, 20 μL of cell suspension and 80 μL of pre-warmed 0.5% *w/v* low-melting agarose in PBS were mixed. Then, cell suspension was layered on the pre-coated microscope slides with 1% *w/v* normal-melting agarose, and the slides incubated at 4 °C for 30 min for solidification. Lysis at 4 °C for 2 h followed in pre-cooled lysis buffer (2.5 Μ NaCl, 100 mM EDTA, 10 mM Tris base, NaOH was added to pH 10.0, 1% Triton X-100 freshly added). DNA unwinding for 20 min in cold-fresh electrophoresis buffer (300 mM ΝaOH, 1 mM EDTA, pH > 13) followed and electrophoresis (25 V/300 mA) was performed at 4 °C for 20 min. Neutralization was followed, in cold Neutralization Buffer (0.4 M Tris–HCl, pH 7.5) for 2 × 15 min each time, then rinsed in dH_2_O for 5 min and the fluorescent dye SYBR Green I is used for staining in a dilution of 1:10,000 in TE buffer (10 mM Tris-HCl, 1 mM EDTA, pH 7.5). The slides were stored at 4 °C until observation under a fluorescent microscope (Olympus BX53, Tokyo, Japan) at 40× magnification.

Results were obtained from 100 randomly selected cells from two independent experiments (50 cells/experiment), where duplicate slides were used for each sample. Tail parameters were then calculated automatically using the CaspLab–Comet Assay Software Project. The tail parameters used in this study were the tail moment (TM), the tail length (TL) and the % DNA tail (TD). The TM was defined by Olive et al., (1990) as the amount of DNA in the tail and the mean distance of migration in the tail [30].

### 2.6. Statistical Analysis

Data from XTT and Comet assays were analyzed using one-way ANOVA followed by Tukey’s multiple pair wise comparisons. Data from flow cytometry and TBARS were analyzed by Mann-Whitney t-test. Data for in vitro cell-free methods are presented as mean ± SD, while for cell based methods as mean ± SEM. GraphPad Prism software version 8.0.1 was used (GraphPad Software, San Diego, CA, USA). The significance level was set at *p* < 0.05 (* *p* < 0.05; ** *p* < 0.01; *** *p* < 0.001; † *p* < 0.0001). All the experiments were carried out in triplicate and at least on two (cell-free) or three separate occasions (cells).

## 3. Results

### 3.1. Brine Extract Characterization

**Figure 1:**
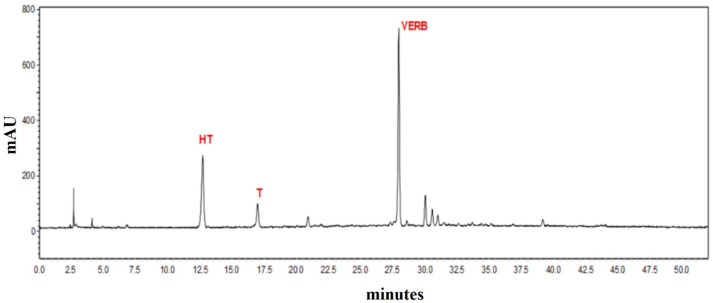
HPLC-DAD chromatogram obtained from the analysis of TOWW extract, applying the IOC proposed method. Hydroxytyrosol (HT), Tyrosol (T) and Verbascoside (VERB) are highlighted.

Table 1 and HPLC-DAD chromatogram in Figure 1 show the main phenolic components, determined in the brine extract of Kalamon olives. The main metabolite of the extract was HT, with a content of 7.5 mg/100 g of extract, followed by VERB with a content of 6.5 mg/100 g of extract and then T with a significantly lower concentration level of 2.9 mg/100 g of extract.

### 3.2. Free Radical Scavenging Activity of the TOWW Extract

**Figure 2:**
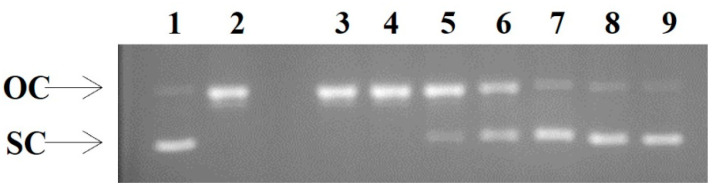
Protective activity on ROO^•^-induced oxidative damage. Lane 1: pBluescript-SK+ plasmid DNA without any treatment; Lane 2: plasmid DNA exposed to ROO• alone; Lane 3–8: plasmid DNA exposed to ROO• in the presence of increasing concentrations of brine extract (Lane 3: 6.25 μg/mL; Lane 4: 12.5 μg/mL; Lane 5: 25 μg/mL; Lane 6: 50 μg/mL; Lane 7: 100 μg/mL; Lane 8:200 μg/mL); Lane 9: plasmid DNA exposed to the maximum tested concentration of the extract alone (200 μg/mL). OC: open circular; SC: supercoiled (representative figure of the three experimental repeats).

Table 2 summarizes the IC_50_ values of the TOWW extract, which exhibits strong antiradical activity in all the tested assays compared to positive controls. It is known that the lower IC_50_ value, the higher the antioxidant activity is. Remarkable is the fact that the extract not only neutralizes the artificial radicals, DPPH^•^ [IC_50_: 19.62 μg/mL vs. Ascorbic Acid (AA) IC_50_: 4.0 μg/mL] and ABTS^•+^ (IC_50_: 5.19 μg/mL vs. AA IC_50_: 3.0 μg/mL), but also has a very high ability to neutralize superoxide radicals (O_2_^•−^) (IC_50_: 77.01 μg/mL vs. Ellagic Acid IC_50_: 255.0 μg/mL), which are produced during normal metabolism, but in high levels can cause DNA and tissue damage, leading to various diseases [31]. Furthermore, the brine extract exerted strong ability to reduce iron Fe (III) into Fe (II) (AU_0.5_: 10.94 μg/mL vs. AA AU_0.5_: 5.0 μg/mL), which further reinforced the antioxidant ability of the extract. Finally, the brine extract exhibited protective activity against ROO^•−^ induced DNA plasmid breakage (Figure 2), with much lower IC_50_ value (IC_50_: 48.49 μg/mL) than the positive control (AA) (IC_50_: 300.0 μg/mL).

### 3.3. Biological Activity of the Brine Extract

#### 3.3.1. Assessment of Non-Cytotoxic Concentrations of the Extract

**Figure 3:**
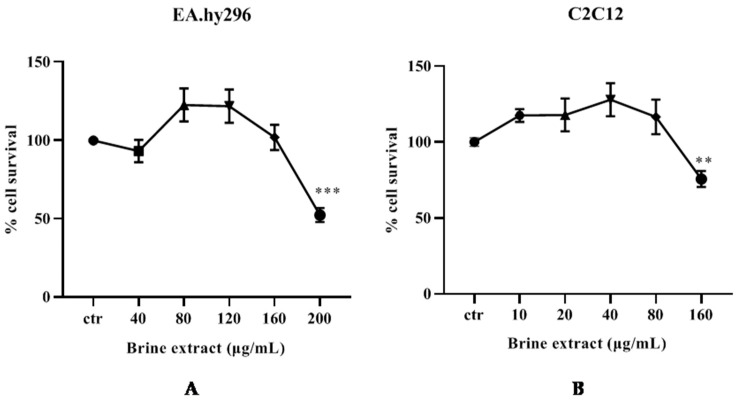
Cell viability under increasing concentrations of brine extract of (**A**) EA.hy296 cells and (**B**) C2C12 cells. Cells were treated in the absence or in the presence of brine extract for 24 h, in serum free medium. Data represents the mean of three independent experiments performed in triplicate and expressed as % change to the untreated cells (ctr) ± SEM.** *p* < 0.01; *** *p* < 0.001indicated a statistically significant difference between treated samples and the control.

Initially, the non-cytotoxic concentrations of the extract were investigated, in order to be used in the continuation of the antioxidant activity experiments. Cells were treated under increasing concentrations of the brine extract for 24 h, in serum-free medium [20,26,32] and cell viability was estimated using the XTT assay. Cytotoxic concentration was defined as the concentration, where a statistically significant decrease in cell viability was observed (Figure 3). According to the results, for EA.hy926 and C2C12 cells, 40–160 μg/mL (Figure 3A) and 10–80 μg/mL (Figure 3B) of brine extract, respectively, were used as non-toxic concentrations at the following experiments.

#### 3.3.2. Estimation of ROS, GSH and TBARS Levels

**Figure 4:**
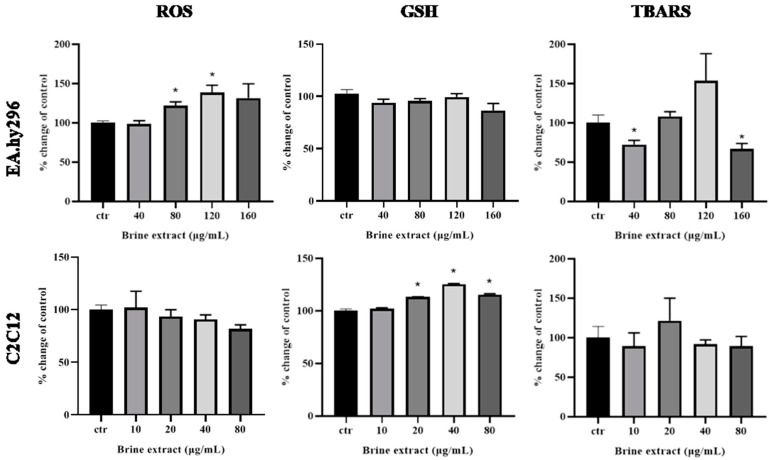
ROS, GSH and TBARS levels after 24 h exposure at increasing concentrations of brine extract. All results are expressed as the mean ± SEM of at least three independent experiments. * *p* < 0.05 indicated a statistically significant difference between treated samples and the control.

ROS, GSH and TBARS levels are well established biomarkers for the assessment of the antioxidant activity of polyphenolic compounds, at non-cytotoxic concentrations, in cell lines [33]. To that end, EA.hy296 and C2C12 cells were treated with 40–160 μg/mL and 10–80 μg/mL of brine extract, respectively, for 24 h, in serum-free medium (Figure 4). Data showed that ROS levels were not significantly altered at any concentration in both cell lines, except in case of 80 and 120 μg/mL treatment of EA.hy296 cells, where a statistically significant increase was observed (Figure 4). GSH levels were unchanged at EA.hy296 cells, while a statistically significant increase under treatment with 20–80 μg/mL of brine extract was observed at C2C12 cells (Figure 4). Of particular interest was the change in TBARS levels at EA.hy296 cells, as a statistical significant decrease was observed after exposure at the lower concentration of brine extract (40 μg/mL), following by an increase at 120 μg/mL, where ROS levels were enhanced, simultaneously, while a reduction was observed at the highest concentration (160 μg/mL). However, at C2C12 cells no statistically significant alteration was noticed (Figure 4).

#### 3.3.3. Geno-Protective Ability of Brine Extract under Oxidative Conditions

Next, we investigated the ability of brine extract to protect DNA from oxidative damage, using Comet assay. For this purpose, the concentration of brine extract chosen was 40 μg/mL, where an increase at GSH levels at C2C12 cells and reduction in TBARS levels at EA.hy296 cells were observed (Figure 4). Both cell lines were pre-treated for 23 h with or without 40 μg/mL of brine extract, following by incubation for another 1 h in the absence or presence of 250 µM H_2_O_2_, as oxidative agent. After the 24-h incubation period, 100 randomly selected cells for each sample were analyzed, usingthe CaspLab–Comet Assay Software Project. From this analysis, three parameters were calculated automatically: the tail moment (TM), the tail length (TL) and the % DNA tail (TD).

**Figure 5:**
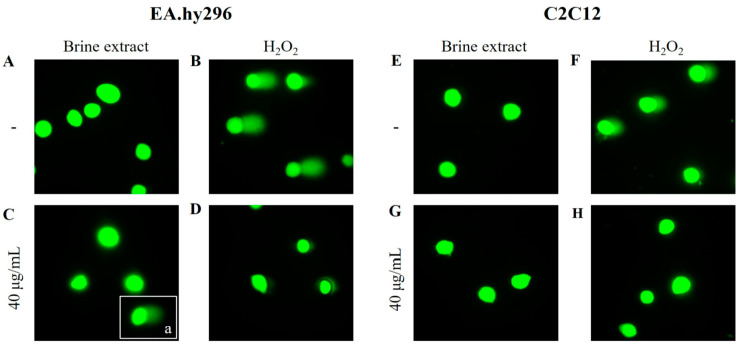
H_2_O_2_-induced DNA damage was prevented after cell pre-treatment with brine extract. Representative images of Comet assay after treatment with 40 μg/mL of brine extract in the absence or presence of 250 μΜ H_2_O_2_, at EA.hy296 cells (**A**–**D**) and at C2C12 cells (**E**–**H**). (**A**,**E**): untreated cells (control), (**B**,**F**): cells treated with 250 μΜ H_2_O_2_ for 1 h, (**C**,**G**): treated cells with 40 μg/mL of brine extract for 24 h, C.a: representative figure of cell subpopulation (12%) with DNA damage in the presence of brine extract, because we were unable to have one representative image that contains both types of cells (with and without tail), thereby we showcase an additional figure as an inset inside the figure; (**D**,**H**): pre-treated cells with 40 μg/mL brine extract for 23 h followed by incubation with 250 μΜ H_2_O_2_ for 1 h.

Figure 5 shows representative images of each sample, where the comet tails depended on the treatment, with longer tails indicating more DNA damage. Untreated cells (control) showed no tails in either cell lines (Figure 5A,E). In contrast, treatment with 250 μM H_2_O_2_, for 1 h, caused significant DNA damage, as evidenced by the comet tails in both cell lines (Figure 5B,F). Surprisingly, the pre-treatment with 40 μg/mL of brine extract protected from the DNA damage caused by H_2_O_2_, as comet tails were significantly decreased or disappeared in both cell lines (Figure 5D,H).Cells incubated with brine extract alone, appeared similar to untreated cells (Figure 5C,G). However, in case of endothelial cells, a small number of cells (12%), had comet tails in the presence of brine extract alone (Figure 5C(a)), indicating a cell type specific response.

**Figure 6:**
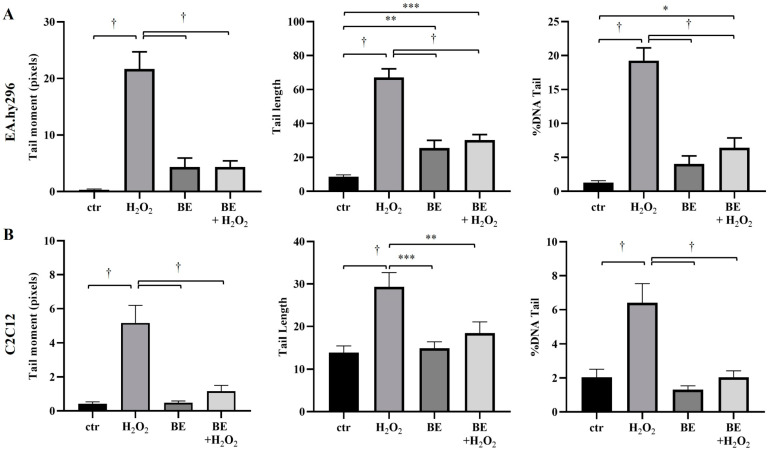
Results from automatic calculation of the three parameters tail moment (TM), tail length (TL) and % DNA tail (TD), using the CaspLab–Comet Assay Software Project at (**A**) EA.hy296 cells and (**B**) C2C12 cells. In both cases, cells were pre-treated or not with 40 μg/mL of brine extract (BE) for 23 h. Then 250 μM H_2_O_2_ was added for 1 h treatment alone or in the presence of BE, while untreated cells were the control sample. Next, a Comet assay was performed. Data were obtained from 100 randomly selected cells from two independent experiments (50 cells/experiment), where duplicate slides were used for each sample, and presented as mean ± sem. * *p* < 0.05 ** *p* < 0.01; *** *p* < 0.001; † *p* < 0.0001 indicated a statistically significant difference between treated samples and the control.

These observations were, also, verified by the automatic quantification of the three parameters TM, TL and TD (Figure 6, Tables S1 and S2). More specifically, according to the three parameters, the H_2_O_2_-induced DNA damage was greater at endothelial cells (EA.hy296) (Figure 6A, Table S1), than in myoblasts (Figure 6B, Table S2). However, this damage was returned at the same levels of brine extract alone for endothelial cells (Figure 6A, Table S1) or at control levels for myoblasts (Figure 6B, Table S2). In conclusion, pre-treatment with brine extract (40 μg/mL) protected endothelial cells and myoblasts from DNA damage caused by oxidative agent (H_2_O_2_).

## 4. Discussion

The present study, for first time, demonstrates a holistic approach for the in vitro antioxidant capacity of an extract, originated from brines after Kalamon olive fruits debittering process, according to Greek style, as well as its ability to protect from oxidative DNA damage.

The phenolic composition of Table Olive Wastewater is quite variable as it directly depends on many factors such as the geographical region, harvest period, olive tree variety, as well as the debittering method [34]. In this study, the results of the HPLC-DAD analyses showed that the brine extract of Kalamon olives was rich in HT, VERB and T. Specifically, the main metabolite of the extract was HT, while the T content was at much lower levels. This observation is in agreement with previous studies in by-products of table olives of different varieties [15,16], as well as of Kalamon olives [13,14]. The increased amount of HT in brine of edible olives is due to the hydrolysis of oleuropein, which is the major phenolic component in fresh olive fruits and is responsible for their bitter taste [35]. Furthermore, T derived from the hydrolysis of Ligstroside, a phenolic glycoside found in the olive fruit, during the debittering process [36,37].

Verbascoside was another biophenol determined in the brine extract, which has also been detected in brines from Greek-style debittering process of Kalamon olives, according to previous studies [13,14]. It has been observed that the concentration levels of VERB in TOWW by-products of Kalamon olives are directly dependent on the stage of olive ripening [38].

Therefore, as TOWW has been highlighted as a rich source of polyphenols, the antioxidant capacity of the enriched extract was evaluated, using free radical scavenging assays. The brine extract exhibited a lower IC_50_ value in ABTS^•+^ (5.19 μg/mL) than in DPPH^•^ assay (19.62 μg/mL), which may be ascribed to the different solvents using in the two assays [39]. In ABTS^•+^ assay the solvent is water, while in DPPH assay the solvent is MeOH [40]. As a result, the brine extract, which is rich in HT and VERB, two highly hydrophilic components [41,42], exerted higher scavenging activity in ABTS^•+^assay. Τhis change has also been observed in a previous study investigated olive extracts, rich in polar molecules [20]. Τo these data, we highlighted the antioxidant capacity of the brine extract, using another two in vitro assays, O_2_^●−^ and RP. The brine extract not only exerted great ability to neutralize the artificial radicals, DPPH^•^ and ABTS^•+^, but had, also, strong ability to scavenge superoxide radicals (O_2_^•−^) (IC_50_: 77.01 μg/mL vs. Ellagic Acid IC_50_: 255.0 μg/mL), an essential signaling molecule for the life of aerobic organisms, butat toxic levels could cause DNA damage [43,44].

There are not many data evaluating the antioxidant capacity of brine extracts. However, in the available data, the IC_50_ values at in vitro assays range at the same levels as ours [16] or much higher [15,17]. These changes may be associated with many factors such as, the olive cultivar, olive fruit maturation, the physicochemical characteristics of brine samples or storage conditions. For example, estimation of antioxidant capacity of brine extracts from Moroccan Piccholine olives indicated much higher IC_50_ values, using DPPH^•^ and RP assays compared to our findings [15]. Another study has shown that, the antioxidant ability of brines is depended on the maturity of the olive fruit, with black and purple-olive brines, exhibited higher antioxidant potential due to higher concentration of phenolic compounds [17].

The three main phenolic compounds of the brine extract, HT, VERB and T are known for a variety of biological activities including antioxidant, anti-cancer, anti-inflammatory, neuroprotective and cardioprotective effects [45,46,47]. These beneficial health effects of phenolic compounds are related to their bioavailability and metabolism. There are several studies which have shown the bioavailability of these phenolic compounds using in vitro systems. Based on the existing data, HT is well absorbed across intestinal epithelial cell monolayers [48], even though there is a better absorption of its lipophilic derivatives, which converted into free HT [49]. Furthermore, extensive uptake and metabolism of HT was observed at HepG2 cells, as an in vitro model for human liver [50,51]. Additionally, there are several in vivo studies which have shown that HT bioavailability depends on human physiology, as well as on the physicochemical characteristics of the food matrix. In particular, HT bioavailability is increased in oily matrices [52], while sex is another factor which determines HT bioavailability, maintaining this component in the bodies of female rats for a longer period, due to a differential metabolism and enterohepatic circulation [53]. Furthermore, there is a study which supports increased levels of HT in urine samples, when it is administered as red wine, probably due to the interaction between ethanol and dopaminergic ways [54]. Finally, there is a very good distribution ability of HT and its metabolites in many tissues such as muscle, testis, liver, and brain, while it is accumulated in kidney and liver, thus it can exert its health beneficial effects [55]. Regarding T, less data are available. Despite the difference of T in one hydroxyl group compared to HT, their bio availabilities are quite dissimilar. In comparison to HT, T is less prone to biotransformation and although it has an efficient permeability, there are fewer metabolic reactions, possibly due to the lack of extra hydroxyl [56]. Regarding the bioavailability of VERB there is limited information. A study using Caco2 human intestinal epithelial monolayers has assessed the digestive stability and intestinal uptake of VERB, using an in vitro digestion/Caco-2 model system [57]. Next, the same scientific group found rapid absorption of purified VERB using normal colonic mucosal sections from 11 individual patients, however, its accumulation efficiency was very low [58].

According to our results, the strong antioxidant capacity of our extract could be due to the high content in HT and VERB in black Kalamon olive fruits, which were debittered. The phenolic compounds exert their antioxidant activity by acting as free radical scavengers, hydrogen donators, metal chelators and singlet oxygen quenchers. However, they could act as pro-oxidants (electron acceptors) under specific conditions [59]. The ability of polyphenols to exert their pro-oxidant/antioxidant effects depends on their concentration and structure [20,60,61]. It has been reported that HT exerts pro-oxidant effects, due to its iron- and copper-reducing activities. These reduced metals can catalyze the production of OH• radicals by the Fenton reaction [20]. Pro-oxidant effects have been, also, demonstrated for VERB due to its high concentration and prolonged incubation period [62].

Our study, for the first time, demonstrates the biological effect of a brine extract in redox status markers, at cellular level. For this purpose, two different cell lines were selected: EA.hy926 endothelial cells and C2C12 murine myoblasts. It is known that the vascular endothelium and muscle tissue are vulnerable tissues at oxidative stress. An imbalance of ROS generation and antioxidant defense mechanisms in endothelium represents early stages of vascular damage, which finally lead to various human diseases [32,33]. The muscle tissue is exposed to high concentrations of ROS under physiological conditions, such as exercise [34]. However, oxidative stress reduces the differentiation of myoblasts, the precursor cells of skeletal muscles, and induces their apoptosis, thus leading to disturbance of muscle development and differentiation, a fact that can cause muscle disorders [35,36]. Therefore, EA.hy926 and C2C12 cell lines were used as typical cell models for the investigation of the impact of oxidative stress on vascular endothelium and the ability of muscle regeneration [37,38,39,40,41].

Using flow cytometry analysis for detection of ROS and GSH levels, after treatment at non-cytotoxic concentrations of the brine extract, a statistically significant increase at GSH levels and a simultaneous trend of reduction in ROS levels were observed only at myoblasts (Figure 4). On the other hand, in the presence of the brine extract, ROS levels increased significantly while GSH levels did not affected at endothelial cells (Figure 4). Thus, the effect of brine extract on ROS and GSH levels seemed to be cell type dependent. It has been proposed that, different cell response regarding the GSH levels could be justified by different expression levels of genes coding enzymes implicated in GSH synthesis (GCLM gene), or by the different signaling pathways mediated for redox homeostasis according to the cell type [63]. More specifically, GSH synthesis and metabolism controlled by intercellular cell adhesion molecule-1 (ICAM-1) in endothelial cells, under basal and inflammatory conditions [64,65], while ICAM-1 molecule is not expressed at C2C12 cells [66]. Furthermore, it is known that HT increases GSH levels by the activation of the transcription factor nuclear factor (erythroid-derived-2)-like 2 (Nrf2), which is a key regulator of the antioxidant defense mechanism [67,68,69]. On the other hand, HT decreases ICAM-1 expression levels [70], which could explain why the brine extract did not induce GSH levels at endothelial cells, in our study. Combining the aforementioned data, a proposed mechanism is that HT of brine extract reduces ICAM-1 expression levels at endothelial cells, as a result to prevent GSH synthesis through this pathway, while at myoblasts, that did not express ICAM-1 molecule, HT induces GSH levels through a different signaling pathway.

The antioxidant capacity was further investigated in cells, measuring lipid peroxidation levels (TBARS). Interestingly, a biphasic dose–response characteristic change was observed at endothelial cells (Figure 4), where at low dose of brine extract (40 μg/mL) the reduction in TBARS levels indicates protective effect for cells while TBARS levels exerted a trend of increase at higher dose (120 μg/mL). At this concentration (120 μg/mL) the simultaneous increased ROS levels advocate a toxic effect, which is reset at control levels, at the higher concentration of brine extract (160 μg/mL). These results demonstrate a hormetic response triggered by brine extract at endothelial cells, a phenomenon that has been observed and described for olive oil polyphenol extracts [26,32] and other natural and chemical agents, as a model of adaption followed by different cell types under a stressful stimulus [71].

Next, we tested the ability of brine extract to protect from DNA damage at oxidative conditions using two different approaches: (a) in vitro investigation of plasmid protection from damages induced by AAPH and (b) at cellular level using Comet assay. In both cases the brine extract had the ability to protect from DNA damage under oxidative conditions. In first case, the brine extract was much more bioactive than ascorbic acid (AA), as indicating by IC_50_ values (IC_50_: 48.49 μg/mL vs. IC_50_: 300 μg/mL AA) (Table 2).

In order to investigate the geno-protective effect in cells, we performed a COMET assay. This method is a well-established and well accepted molecular method, for the evaluation of DNA damage and repair in various cell types and tissues, which detects both single stranded and double stranded DNA breaks and alkali-labile sites [72,73]. For this purpose, we selected the 40 μg/mL concentration of extract, in which a protective role at both cell lines was observed: increased GSH levels at C2C12 cells and reduced TBARS levels at EA.hy296 cells. We found that the pre-treatment with brine extract protected from the H_2_O_2_-induced DNA damage in both cell lines, as comet tails significantly reduced or absent in the presence of extract in comparison to H_2_O_2_-treated cells (Figure 5 and Figure 6). H_2_O_2_ causes DNA strand breaks by generation of the hydroxyl radicals (OH^•^), therefore olive phenols act as free radical scavengers and prevent genomic damages [74].

Despite the fact that there are no corresponding studies in brine extracts, similar geno-protective results have been found, using Comet assay, in the presence of extracts from olive leaves, olive fruits, olive oil and olive mill wastewater, which were attributed to the isolated HT and caffeic acid [72,74,75]. In addition, a recent study has demonstrated the inhibition of paraquat-induced DNA damage by VERB, indicating the geno-protective activity of this component [76]. So, it is possible, the protective effect of our brine extract exerted due to HT and VERB, which are the main biophenolsin the extract.

## 5. Conclusions

In conclusion, this is the first holistic estimation of the antioxidant potential and DNA protective activity from oxidative damage of a brine extract from Kalamon olives debittering, according to Greek style. For this purpose, cell-free and cell-based assays were used. These data highlighted table olive wastewaters from Kalamon debittering as valuable source of bioactive compounds, which could have interesting implications for the development of new products to provide protection and treatment against harmful effects of free radicals.

## Figures and Tables

**Table 1:** The three main biophenols in the brine extract. Data of regression model (linear regression, r-squared and concentration ranges) are given. Data are presented as mean ± SD. The results were expressed in mg analyte per 100 g of extract.

		Linearity of Phenolic Compounds Standards		
Phenolic Compounds	mg Analyte/100 g of Extract	Linear Regression	r^2^	Concentration Range (μg/mL)
HT ^1^	7.5 ± 0.2	y = 84,028x + 39,609	0.9997	0.5–100
T ^2^	2.9 ± 0.1	y = 53,933x − 11,712	0.9987	0.5–100
VERB ^3^	6.5 ± 0.2	y = 71,488x − 50,153	0.9997	10–100

^1^ HT: Hydroxytyrosol. ^2^ T: Tyrosol. ^3^ VERB: Verbascoside.

**Table 2:** IC_50_ and AU_0.5_ values (μg/mL) of brine extract from Kalamon olive fruit debittering. Data are presented as mean ± SD.

	IC_50_ (μg/mL)					AU_0.5_ (μg/mL)
Samples	Solvent	DPPH^•^	ABTS^•+^	O_2_^•−^	ROO^•^	RP
BE	H_2_O	19.62 ± 1.40	5.19 ± 0.36	77.01 ± 5.78	48.49 ± 11.23	10.94 ± 0.54
Positive controls						
Ascorbic acid	H_2_O	4.0 ± 0.2	3.0 ± 0.4	ND	300.0 ± 30.6	5.0 ± 0.1
Ellagic acid				255.0 ±10.4		

## Data Availability

The data presented in this study are available on request from the corresponding author.

## Supplementary Materials

The following supporting information can be downloaded at: https://www.mdpi.com/article/10.3390/antiox12020333/s1, Table S1: Tail parameters of DNA damage using Comet assay in EA.hy296 cells, treated with 40 μg/mL of brine extract in the absence or presence of 250 μM H_2_O_2_. Data are presented as mean ± SEM. Table S2: Tail parameters of DNA damage using Comet assay in C2C12 cells, treated with 40 μg/mL of brine extract in the absence or presence of 250 μM H_2_O_2_. Data are presented as mean ± SEM.
