# Supplementary material for: Antioxidant and DNA-Protective Activity of an Extract Originated from Kalamon Olives Debittering
Source: Antioxidants (Basel). 2023 Jan 31;12(2):333. doi: 10.3390/antiox12020333 (PMC9952268; doi:10.3390/antiox12020333)
Supplement: Supplementary file 1 [file antioxidants-12-00333-s001.zip › Table S1.pdf]

Table S1. Tail parameters of DNA damage using Comet assay in EA.hy296 cells, treated with 40 μg/mL of brine extract in the absence or presence of 250 μM H_2O_2. Data are presented as mean ± SEM.

Treatments	Tail moment	Fold change of the Averages	Tail length	Fold change	%DNA tail	Fold change
control	0.34 ± 0.11	1.00	8.57 ± 1.10	1.00	1.28 ± 0.29	1.00
H_2O_2	21.69 ± 3.02	63.79	67.16 ± 5.08	7.84	19.26 ± 1.87	15.05
BE	4.35 ± 1.60	12.79	25.57 ± 4.44	2.98	4.04 ± 1.17	3.15
BE + H_2O_2	4.33 ± 1.10	12.73	30.30 ± 3.18	3.53	6.42 ± 1.43	5.01

BE: brine extract
