# Supplementary material for: Antioxidant and DNA-Protective Activity of an Extract Originated from Kalamon Olives Debittering
Source: Antioxidants (Basel). 2023 Jan 31;12(2):333. doi: 10.3390/antiox12020333 (PMC9952268; doi:10.3390/antiox12020333)
Supplement: Supplementary file 1 [file antioxidants-12-00333-s001.zip › Table S2.pdf]

Table S2. Tail parameters of DNA damage using Comet assay in C2C12 cells, treated with 40 μg/mL of brine extract in the absence or presence of 250 μM H_2O_2. Data are presented as mean ± SEM.

Treatments	Tail moment	Fold change	Tail length	Fold change	%DNA tail	Fold change
control	0.42 ± 0.11	1.00	13.90 ± 1.57	1.00	2.03 ± 0.48	1.00
H_2O_2	5.16 ± 1.03	12.28	29.33 ± 3.36	2.11	6.40 ± 1.12	3.15
BE	0.48 ± 0.10	1.14	14.92 ± 1.51	1.07	1.31 ± 0.22	0.64
BE + H_2O_2	1.16 ± 0.34	2.76	18.47 ± 2.61	1.32	2.04 ± 0.38	1.01

BE: brine extract
